# Migratory corridor linking Atlantic green turtle, *Chelonia mydas*, nesting site on Bioko Island, Equatorial Guinea to Ghanaian foraging grounds

**DOI:** 10.1371/journal.pone.0213231

**Published:** 2019-06-21

**Authors:** Emily Mettler, Chelsea E. Clyde-Brockway, Shaya Honarvar, Frank V. Paladino

**Affiliations:** 1 Department of Biology, Purdue University, Fort Wayne, Indiana, United States of America; 2 Department of Biology, Purdue University, West Lafayette, Indiana, United States of America; 3 Bioko Marine Turtle Program, Malabo, Equatorial Guinea; Deakin University, AUSTRALIA

## Abstract

This study uses satellite telemetry to track post-nesting movements of endangered green turtles (*Chelonia mydas*) (n = 6) in the Gulf of Guinea. It identifies a migratory corridor linking breeding grounds of Atlantic green turtles nesting on Bioko Island, Equatorial Guinea, to foraging grounds in the coastal waters of Accra, Ghana. Track lengths of 20–198 days were analyzed, for a total of 536 movement days for the six turtles. Migratory pathways and foraging grounds were identified by applying a switching state space model to locational data, which provides daily position estimates to identify shifts between migrating and foraging behavior. Turtles exhibited a combination of coastal and oceanic migrations pathways that ranged from 957 km to 1,131 km. Of the six turtles, five completed their migration and maintained residency at the same foraging ground near the coastal waters of Accra, Ghana until transmission was lost. These five resident turtles inhabit heavily fished waters and are vulnerable to a variety of anthropogenic threats. The identification of these foraging grounds highlights the importance of these coastal waters for the protection of the endangered Atlantic green turtle.

## Introduction

Long distance animal migrations are becoming increasingly well-studied with the advent of reliable, individual-level tracking technology. This technology has produced a more comprehensive understanding of the movements and spatial ecology of marine, terrestrial, and avian species that had previously been difficult to track due to the length of their migrations and inaccessibility of frequently used habitats [1]. The insights gained from these studies can inform policy and management by providing detailed data on species distribution and delineating habitats used during important life history stages, such as breeding, foraging, and nesting areas [2,3]. Animal tracking is often critical in assessing the overlap between human threats and vulnerable wildlife habitats, and therefore can indicate the level of human impact on species that may be otherwise unknown [4,5]. It also gives insight into migration and habitat use patterns across multiple taxa and has revealed behavioral patterns across taxonomically distinct species such as similarities in prey pursuit and predator avoidance behaviors [1]. Tracking animal movements also provides insight into navigation, impacts of food availability and environmental factors on spatial use, and energy costs of different migration patterns, which can improve our understanding of what drives specific movements [6–9].

Satellite telemetry has become one of the most reliable and widely used tracking technologies, especially in marine research. While tracking multiple individuals across many years can reveal population-level shifts in behavior, these sample sizes are difficult to achieve, and smaller sample sizes, particularly in under-studied populations, are not only more feasible, but are critical in identifying previously unknown habitats and observing variations in movement patterns on a smaller scale[1,10].

Satellite telemetry has been used to track the in-water movements and distribution of all seven species of sea turtle [11–17]. It has provided insights into migratory behaviors, locations of foraging grounds and migratory corridors, oceanographic influences on movement patterns, as well as identified locations with high potential for human impact that may contribute to mortality [18–23]. Adult green sea turtles have been known to migrate hundreds to thousands of kilometers between nesting seasons [13,24,25]. Green turtles typically show fidelity to foraging grounds and post-nesting migratory routes are similar year after year [26]. Consequently, protecting migratory corridors and foraging grounds could have widespread and long-term benefits for entire populations of green turtles [25]. Generally, post-nesting migrations are direct movements to foraging habitats, with little energy spent on detours [24,26,27]. However, a number of studies have shown plasticity in migratory behavior among green turtles traveling toward similar destinations, with some individuals taking indirect routes, including both open ocean and coastal pathways, while other individuals of the same population take more direct routes [28,29].

Since in water habitats come with a variety of unique threats, including resource mining, fishing, and anthropogenic pollution, understanding oceanic habitat use and migration patterns is imperative to designing effective marine conservation strategies [30–33]. Green sea turtles have been classified as endangered by the IUCN since 1982, however despite their international protection and conservation status, they are highly threatened by intentional harvest and incidental bycatch in fisheries [34]. Both of these threats are common in the Gulf of Guinea, intentional harvest occurs from both in-water habitats and nesting beaches, and green turtle bycatch occurs in both small-scale and industrial fishing operations [31,35,36]. Oil and gas development has also rapidly intensified in the Gulf of Guinea in recent years [37], and poses diverse, but difficult to measure, threats to sea turtle populations, with an increase in channel dredging, ship traffic, oil leaks, and chemical pollution, which can affect adult turtles that forage or travel close to offshore platforms [33]. These threats highlight the need to study migration patterns and foraging ground locations of sea turtles to better understand their vulnerabilities.

Bioko Island, Equatorial Guinea is home to the second largest nesting rookery for green turtles in Africa, and as such studying this population could have widespread benefits for green turtles throughout the entire region [38–40]. Current estimates of this population range from 454–649 nesting females/year; however it has seen an estimated 78% decline since the 1940’s [34,40]. Despite this, little is known about the in-water habitats and behavior of green turtles in the Gulf of Guinea. Green turtles that were flipper tagged on Bioko Island, Equatorial Guinea, in 1996–1998 have been recaptured in waters off the coast of Ghana, at least 1250 km from the nesting beaches of Bioko, in Corisco Bay, Gabon, about 280 km from Bioko, and off the coast of southern Gabon, at least 760 km from Bioko [41]. Since then, there have been no studies on post-nesting migration routes of green turtles from Bioko, and only one in West Africa, in which satellite telemetry was used to track green turtles nesting in Guinea-Bissau [42].

To address the lack of knowledge on the post-nesting migratory routes of Atlantic green turtles in the Gulf of Guinea, we used satellite telemetry to track turtles from a nesting beach along the southern coast of Bioko Island. Our specific objectives were to (1) map the post-nesting migration routes of green turtles from Bioko Island, (2) determine the directness of migratory routes and identify migratory corridors in the area, (3) categorize these migratory routes as coastal, open ocean, or both, and (4) locate coastal foraging grounds.

## Materials and methods

### Ethics statement

This study was carried out in accordance with all federal, international, and institutional guidelines. All data was collected under the protocol approved by the Purdue Animal Care and Use Committee (PACUC Protocol Number 1410001142). Permissions to work within the protected area and with the study species were granted by the Instituto Nacional de Desarrollo Forestal y Gestión del Sistema de Áreas Protegidas (INDEFOR-AP permit #227), and the research protocol was approved by the Universidad Nacional de Guinea Ecuatorial (UNGE permit number 1011191091017).

### Study site

**Fig 1:**
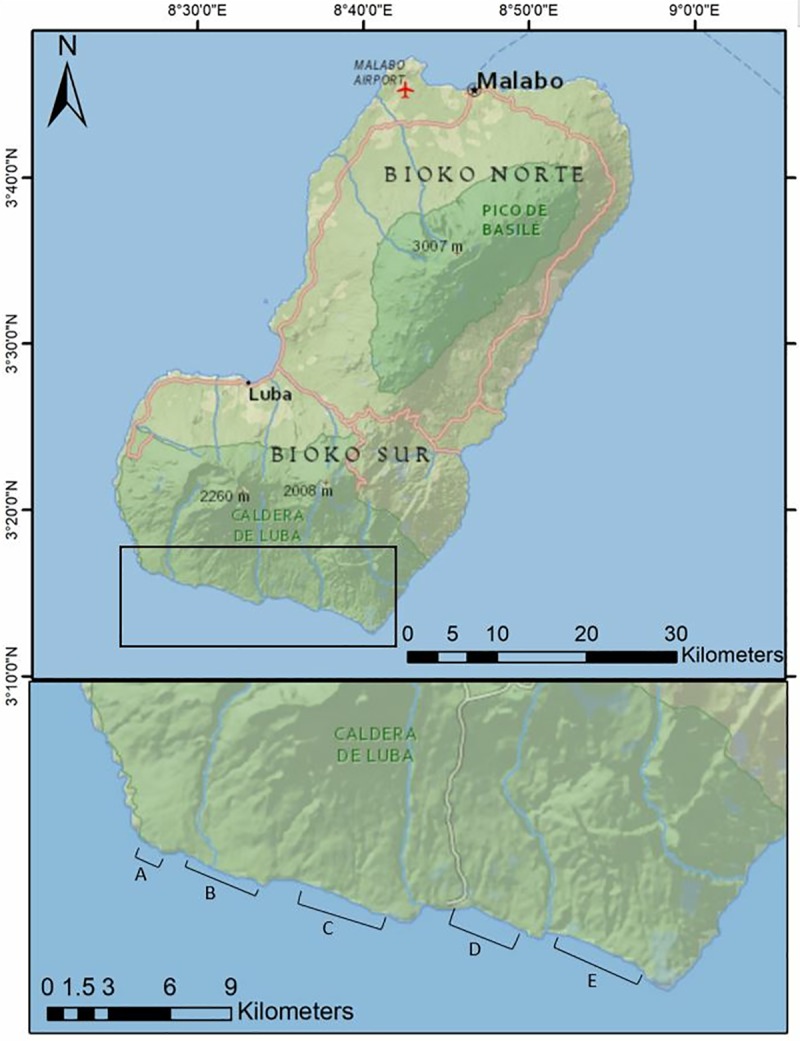
Map of the sea turtle nesting beaches on Bioko Island, Equatorial Guinea. Gran Caldera Southern Highlands Scientific Reserve is shown in dark green covering the southern third of the island. Insert shows the five nesting beaches (A-E) in relation to the nearest village, Ureca. Satellite transmitters were attached to green turtles nesting on Beach C, at the end of the nesting season in January-February 2018. Service Layer Credits: National Geographic, Esri, Garmin, HERE, UNEP-WCMC, USGS, NASA, ESA, METI, NRCAN, GEBCO, NOAA, increment P Corp.

Bioko Island, Equatorial Guinea (2027 km^2^) is situated 175 km Northwest of mainland Equatorial Guinea. The southern coast has approximately 20 km of black sand beaches suitable for sea turtle nesting, all of which are within the legally protected Gran Caldera and Southern Highlands Scientific Reserve (Fig 1). The remainder of Bioko’s 150 km coastline is generally unsuitable for sea turtle nesting due to cliffs, rocky beaches, and proximity to villages and roads [38]. Four species of sea turtles (leatherback, *Dermochelys coriacea*; green, *Chelonia mydas*; olive ridley, *Lepidochelys olivacea* and, hawksbill, *Eretmochelys imbricata*) nest across the five nesting beaches (8°66’-8°46’ E and 3°22’-3°27’ N), with the largest numbers of green turtle nests on beaches A, B, and C [40].This study was conducted on Beach C, chosen for its accessibility and high densities of green turtles (Fig 1).

### Turtle selection

Nesting season for green turtles on Bioko spans October through February [38]. Satellite transmitters were attached at the end of nesting season, in order to focus on tracking post-nesting migration and locational data from foraging grounds. Turtles that had laid their last nest, and therefore did not have developing vitellogenic follicles when scanned with a portable ultrasound (SonoSite 180 Plus; FUJIFILM SonoSite, Bothell, WA, USA), were preferentially selected as this generally indicates that the turtle is about to begin the post-nesting migration [17]. In addition, only turtles that had finished nesting and seemed to be in good health without any scarring or damage to the carapace where the transmitter would be attached were selected. Individuals were identified using a unique injectable passive integrated transponder (PIT) tag (AVID Identification Systems Inc., Norco, CA).

### Satellite transmitters

In January and February, 2018, six satellite transmitters (SirTrack, Kiwisat 202; Sirtrack, Havelock North, New Zealand) were attached to green turtles on Beach C, Bioko Island, after they had finished nesting. The transmitters were attached following the methods developed by Balazs et al. [43] modified by Luschi et al. [24], Troeng et al. [44], and Seminoff et al. [21]. Specifically, the carapace was cleaned, first with water, then with alcohol, and then scored with sandpaper to increase the strength of attachment. Transmitters were attached using Powers Pure50+ Two-Component Epoxy Adhesive (Powers, Brewster, NY, USA) to secure each transmitter to the second central scute of the carapace. Each turtle was restrained by a team of four or five researchers, and a wet cloth placed over the turtle’s eyes, to keep each turtle calm and in place while the epoxy hardened.

### Movement analysis

Location data was relayed via the Argos satellite system, and location points were filtered using the “argosfilter” package for R (R statistical software, R 3.4.3, Vienna, Austria), which removed any point that required a travel speed >5 km/hr [24]. The filtered location data was fit with a state-space model using the ‘bsam’ package [45] for R to estimate the behavioral state of the turtles. Filtered locational data was used instead of raw data to enhance the accuracy of the state space model [46]. The ‘bsam’ package, based on the Bayesian switching state space model developed by Jonsen et al. [47] was applied to the turtle tracks, using a hierarchical switching first-difference correlated random walk model (hDCRWS). The model was fit with a total of 5,000 Markov Chain Monte Carlo (MCMC) samples after 5,000 were discarded as burn-in, and every 10^th^ sample was retained. This model returns a behavioral mode of 1 (MCMC mean values <1.5) or 2 (values >1.5). Behavioral mode 1 is considered transiting behavior, and behavioral mode 2 is considered area restricted search (foraging) behavior. This model also selects one location per day per turtle to standardize the data across multiple turtles.

Individual tracks were then mapped using ArcGIS 10.2 (Esri, Redlands, CA). Track length and daily travel distance were calculated using R from total track distance. Tracks were overlaid with a map of marine and land Exclusive Economic Zones to show country boundaries [48]. Tracks were also overlaid with ocean surface current data from the Ocean Surface Current Analysis Real-Time (OSCAR) from NASA [49]. OSCAR ocean current estimates use sea surface height, surface vector wind, and se surface temperature to estimate velocity and direction of ocean currents. The estimation model combines geostrophic, Ekman and Stommel shear dynamics, and a complementary term from the surface buoyancy gradient [50]. Current data are provided on a 1/3 degree grid with a 5 day resolution. OSCAR data was downloaded for two consecutive 5-day periods, Feb 10–15 and Feb 15–20, 2018 as this time period captured at least half of all migrations. The data was averaged using ArcGIS, giving a 10-day smoothed resolution. OSCAR data was then scaled linearly on a scale from 0–1 and displayed in ArcMap (ESR, 2009). This data was also visually compared to current data for the same area and time period using NASA’s State of the Ocean data viewer, to ensure that no large variations in ocean currents were lost due to smoothing over a 10-day period (available at https://podaac-tools.jpl.nasa.gov/).

## Results

**Fig 2:**
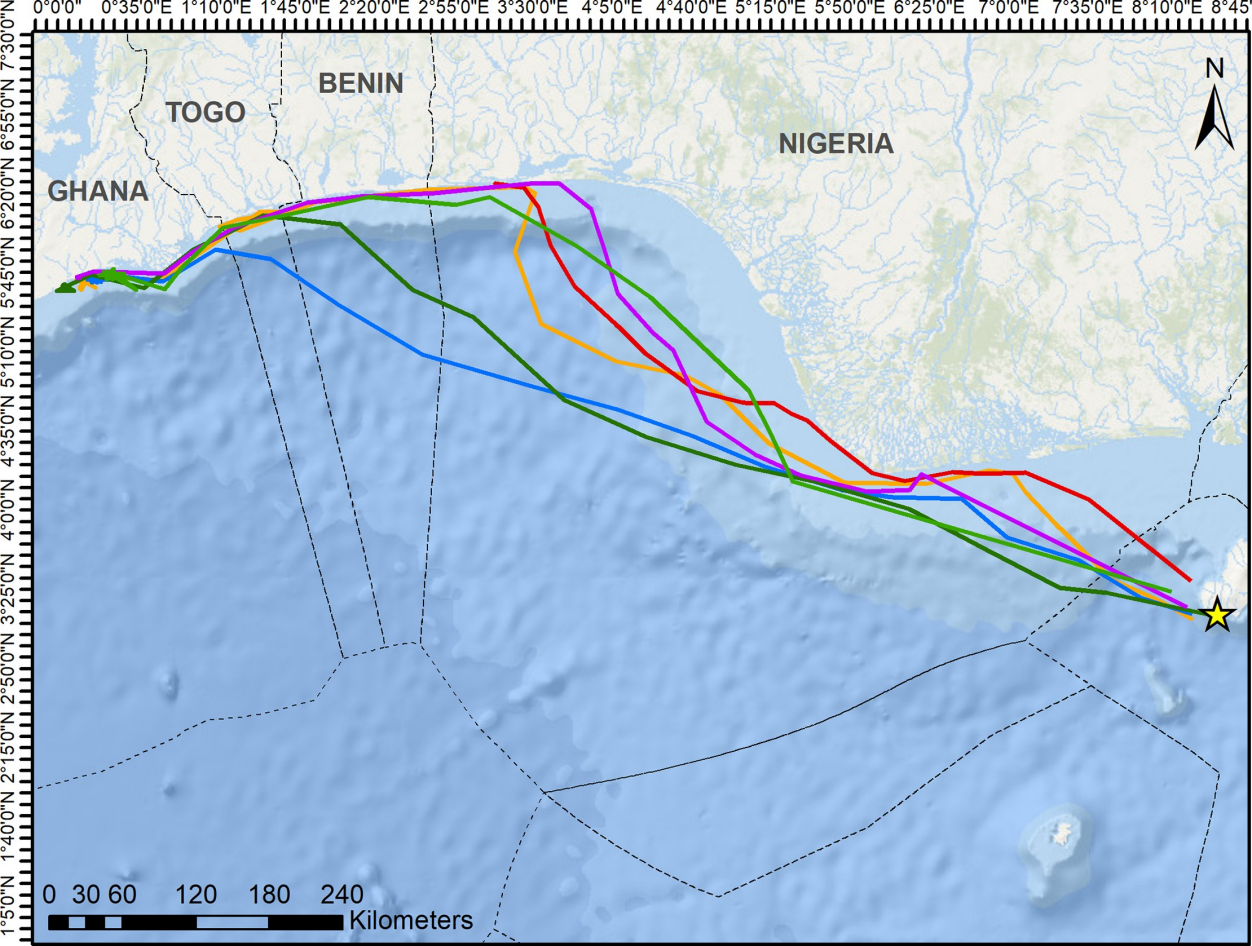
Post-nesting movements of six green turtles (Chelonia mydas) tracked from Bioko Island, after the 2017–18 nesting season. Individuals traveled an average of >1,000km using a combination of oceanic and coastal migratory routes. Two turtles exhibited oceanic migration routes (blue and dark green tracks); the remaining four turtles remained closer to the continental shelf, migrating more directly across the Bight of Benin, to the coastal waters near Lagos, Nigeria, and then maintained a coastal route. Dotted lines represent the Exclusive Economic Zones (EEZs) of each country. Service layer credits: Esri, Garmin, GEBCO, NOAA NGDC, and other contributors Esri, HERE, Garmin, OpenStreetMap contributors, and the GIS user community.

Tracks were analyzed for a total of 536 days. All turtles (n = 6) began westward migrations, and locational data revealed complete migrations ending in extended foraging behavior (>30 days) for five of the six turtles. Average daily distance traveled was 49.5 km, and the average total distance traveled for these five turtles was 1,055 km. Two distinctly different migratory routes were observed, one oceanic, and the other primarily coastal (Fig 2). Two turtles exhibited oceanic migration routes, spending the majority of migrations over deep water in the pelagic zone. These turtles remained in transit across the Bight of Benin until reaching the coast of Togo and Ghana, where the state space model indicated a switch to foraging behavior. These two turtles migrated for an average of 12.5 days and 989 km, with an average daily speed of 84.4 km/day.

**Fig 3:**
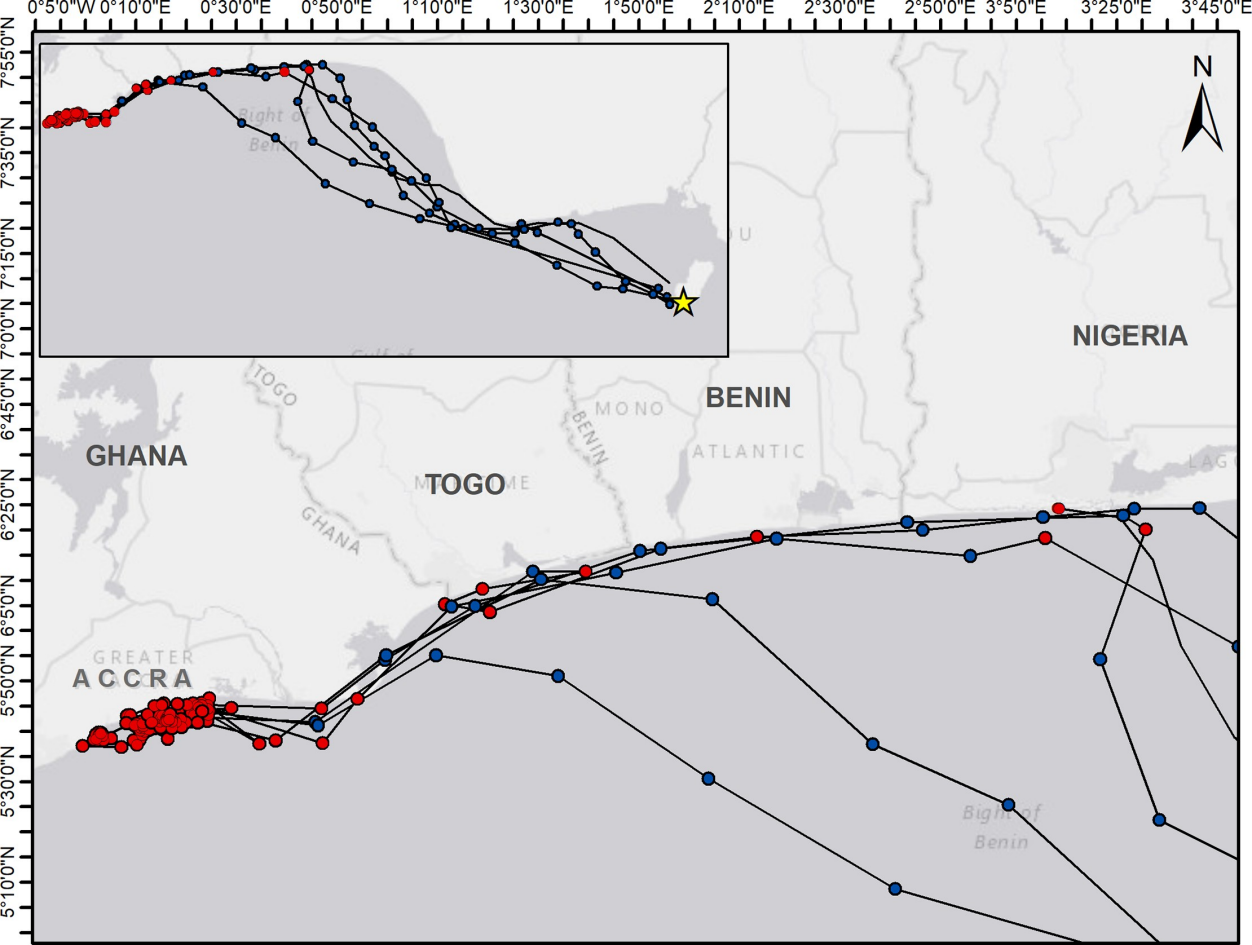
Daily locations (circles) of six turtles tracked from Bioko Island after the 2017–18 nesting season. Blue circles indicate transiting behavior and red circles indicate foraging behavior, as identified by the state space model. Three turtles exhibited migrations interspersed with short (<6 days) periods of foraging, while two exhibit direct migrations, followed by an extended period of foraging. Service Layer Credits: Esri, HERE, Garmin, GEBCO, NOAA NGDC, and other contributors OpenStreetMap contributors, and the GIS user community.

The remaining three turtles that completed migrations used a combination of coastal and oceanic migratory routes, crossing deep ocean basins at times but traveling in the neritic zone for the majority of their migrations. These turtles migrated for an average of 23 days and 1098 km, with an average daily speed of 49.8 before beginning extended foraging activity. These turtles remained closer to the continental shelf, taking a short and direct route across the eastern part of the Bight of Benin, to the coastal waters east of Lagos, Nigeria, and then maintaining a coastal route for the remainder of migrations (Fig 2). These three turtles exhibited short (6 days or less) periods of neritic foraging activity throughout their migrations at suspected stopover foraging habitats off the coasts of Lagos, Nigeria, and Togo and Benin (Fig 3). Turtles exhibited no more than two separate periods of intermittent foraging activity during migrations, and spent up to five consecutive days stopover foraging sites. Most foraging activity was short and isolated, with turtles foraging coastally for one or two days between 3 or more consecutive days of migratory behavior.

**Fig 4:**
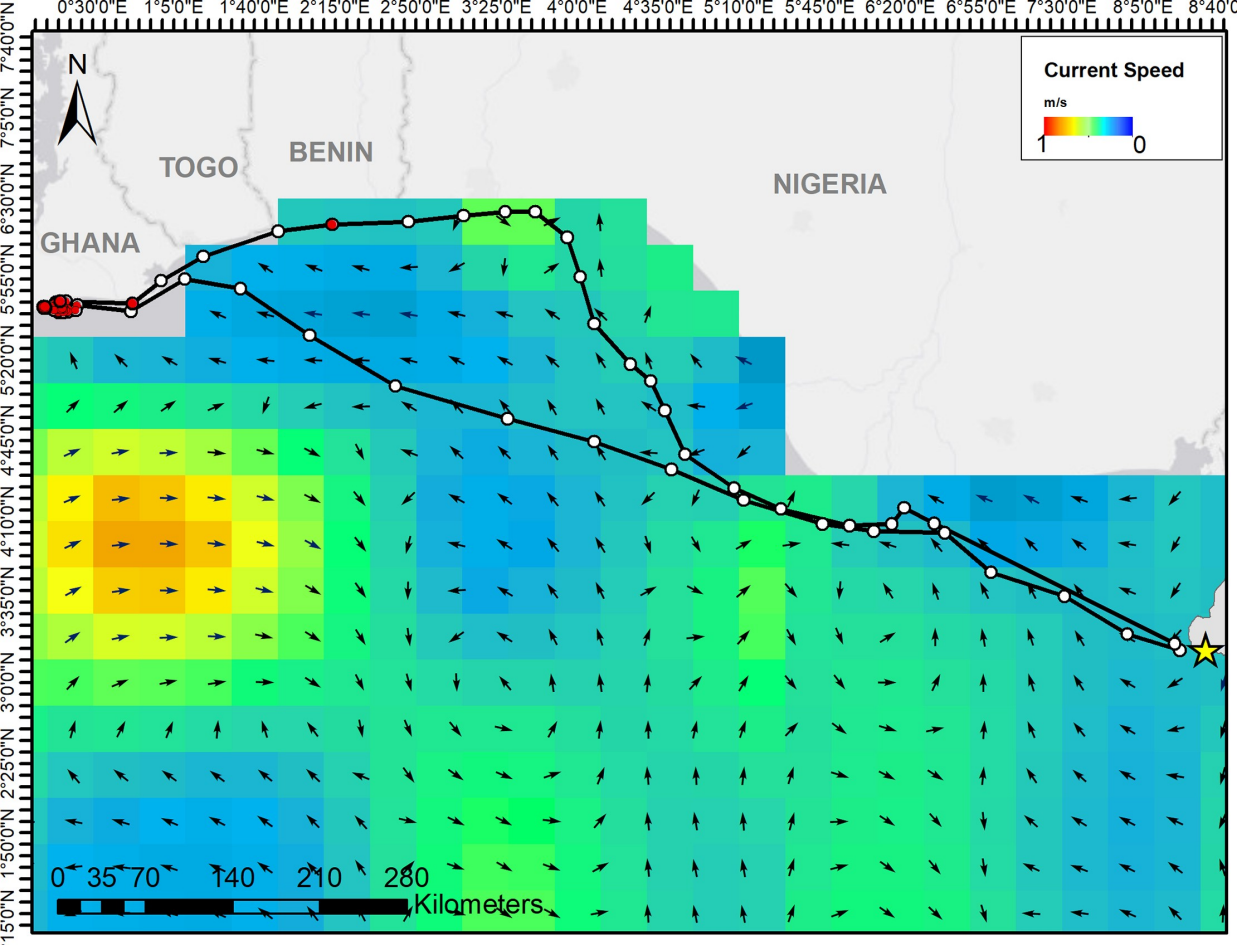
Ocean currents and daily locations (circles) of two green turtles tracked by satellite from Bioko Island across the Bight of Benin. One coastal and one oceanic migration route are overlaid onto averaged ocean surface current data for the 10 day period from 10–20 Feb 2018. White circles represent migrating behavior and red circles represent foraging behavior, as identified by the state-space model. Arrows represent current direction.

Both oceanic and coastal migration routes traveled in accordance with prevailing ocean currents and remained in areas of weak currents for the majority of migrations (Fig 4).

One turtle (Fig 2: purple track) was in transit for 19 days until reaching the coastal waters of Lagos, Nigeria. Beginning on day 20, February 20^th^, all location transmissions were from land. As this turtle had no vitellogenic follicles remaining, there is no evidence that the turtle would have intentionally returned to land, and it is suspected that there was some human interaction that led to the transmitter being moved to land.

All five turtles ultimately began extended periods (>30 days) of residency and foraging behavior off the coast of Ghana, in a 50 km stretch east of Accra and west of the Volta River delta, after migration periods of 14–28 days (Fig 3). While the turtles exhibited both oceanic and coastal migratory routes, all exhibited near-shore foraging activity in shallow (<50 m) waters.

## Discussion

All six turtles migrated westward from Bioko Island, and five turtles completed their migration, ending at a previously undocumented foraging ground in the coastal waters of Ghana (Fig 1). The synchrony in foraging ground destination observed in this study highlights the importance of this habitat for the Bioko population, and suggests that this foraging ground and associated migratory routes probably represent frequently used habitats for this population. However post-nesting movements of Bioko green turtles are not necessarily restricted to the observed migration routes. Previous tag-recapture data suggests that both western and southern migrations occur in this population [41]. The study by Tomás et al. received data from 12 tagged recovered turtles, four of which were found off the coast of Ghana, and the remaining 8 were found near Bioko or south of the island, suggesting that other post-nesting foraging areas most likely exist [41].

Turtles exhibited both oceanic and coastal migration strategies, with two turtles traveling along a shorter route over deeper water (2000-3000m), and three traveling through shallower coastal waters for the majority of their migrations (Fig 1). Variations in migratory routes have been previously observed in green turtles nesting in Tortuguero, Costa Rica, Ascension Island, Brazil, as well as in the Galapagos [21][28][44]. It has been suggested that a coastal migration routes may serve as a navigational tool, allowing turtles to complete migration without the need for direct navigation to a specific destination [28]. Instead of migrating through open-ocean to a foraging ground, which would require more precise navigation, turtles that travel through open-ocean to the mainland coast, and then along the coast ensure that they will reach their destination without the risk of extended searching. Navigation to mainland foraging grounds from nesting beaches on oceanic islands requires complex navigation, a problem which may be solved by open-ocean crossings- which require only a basic compass sense- followed by coastal migrations [28][29].

All of these turtles traveled in the same direction as weak currents during oceanic crossings, and therefore may rely on current direction as an environmental navigational cue when migrating towards a large target, such as the mainland coast. It’s been shown that turtles making similar, but longer, oceanic crossings from Ascension Island may use vector navigation, a simple navigation system of maintaining one direction for a given amount of time, which is possible when migrating in the same direction as ocean or wind currents [51]. Returning to the nesting beaches, a much smaller and more isolated target, however requires more complex and precise navigation. These data contribute to the growing understanding of the complexities of island-finding and the existence of multiple navigational mechanisms used by animals that undergo long-distance migrations.

Long distance migration is associated with high energy cost and all five complete migrations in this study were ~1,000 km. Turtles that used coastal migration routes exhibited short periods of foraging on the way to their final foraging ground (Fig 3). Green turtles are capital breeders, meaning they are generally do not forage during breeding, and therefore are likely to begin post-nesting migrations with depleted energy reserves [52]. The use of stopover foraging sites has been documented in green turtles during coastal migrations in the Mediterranean, Pacific, and South Atlantic, and may decrease the overall energy cost of migration, allowing turtles to rebuild energetic reserves during migration [27][29][53][54]. Utilizing stopovers may be a benefit of a coastal migration pattern, mitigating the longer distance of coastal routes when compared to oceanic routes. It has been suggested that variation in use of stopovers may be driven by individual nutrient levels and metabolic rates, requiring some individuals to make use of stopovers while others can migrate directly, or it may represent “known” sites that offer opportunistic foraging of which other individuals are not aware [53]. In several previously documented cases, these stopovers were within a few days journey from the final destination, and may be discovered during exploratory movements from the final foraging ground [27][29][53].

Turtles migrating from Bioko spent little time at stopovers despite the probable existence of suitable foraging habitat, briefly foraging when advantageous and then continuing to a more distant foraging ground, suggesting fidelity to a specific foraging ground. Reasons for foraging ground selection in sea turtles are largely thought to be due to hatchling dispersal patterns, however degradation of suitable coastal foraging habitat could necessitate longer migrations to more suitable habitat, leading to population-level shifts in foraging ground use [55][56]. Given the existence of nesting populations of green turtles on the beaches nearby this foraging ground in Ghana, and the apparent habitat suitability, it is likely that this foraging ground is used by more than one rookery within the East Atlantic, including those nesting on Bioko Island [56][57].

The discovery of this foraging ground is of particular importance, as only one other foraging ground used by green turtles in the Gulf of Guinea has been documented and protected—Corisco Bay in Equatorial Guinea and Gabon. Yet all five turtles that completed migrations maintained residency in this newly discovered Ghanaian foraging habitat, highlighting the need for protection of this area. Migration routes passed through the exclusive economic zones (EEZs) of five countries (Fig 2), all of which rely heavily on fisheries for economic activity, which poses challenges to regulation and protection of this area. Migrations passed through no marine protected areas (MPAs), meaning throughout the migration pathways and within foraging grounds fishing is unrestricted. Coastal migration routes increase the amount of time turtles spend in shallow, heavily fished coastal waters, and therefore increase the risk of both bycatch and intentional harvest. Direct observations, interviews, and tag returns have shown that green turtles throughout the observed migration route are caught as bycatch in both artisanal and industrial fisheries, in gillnets, driftnets, and purse and beach seines [41][56][58][59]. Data quantifying the extent of bycatch is lacking, however it is suspected that mortality is significant, and is frequently underestimated by studies [60]. One of the six turtles involved in this study had a suspected interaction with humans after only 20 days of migrating, resulting in the transmitter being brought to land. While there is no way of knowing the nature of the interaction, turtles are consistently caught as bycatch in artisanal fishery operations in the area, and there is evidence that once caught, turtles are often transported to land and sold in markets [60].

Furthermore, this Ghanaian foraging ground lies near the outlet of a river that flows past the Kpone power plant as well as the Sakumo Lagoon, an important protected wetland heavily polluted by the inflow of industrial effluent, sewage, and domestic waste [61]. The Sakumo Lagoon has also been shown to have higher than average levels of Cadmium, Cobalt, Copper, Chromium, Nitrogen, and Zinc, which can have toxic effects on marine and aquatic wildlife [61].

## Conclusion

These threats highlight the need for further research into effects of fishing and pollution on this population, as well as the need to protect this valuable foraging habitat. Both industrial and domestic pollution as well as extensive commercial fishing are important issues when considering the protection of this newly discovered foraging ground. The distinct coastal foraging behavior of green turtles lends itself well to protection by spatially-explicit management strategies, such as zonal regulation of fishing and industrial dumping. Protecting nesting beaches in combination with delineating and protecting coastal foraging habitat on a national and multinational level may be key in conserving this highly migratory endangered species.

## Supporting information

S1 Dataset Locational data from transmitter #107904. (XLSX)

S2 Dataset Locational data from transmitter #107905. (XLSX)

S3 Dataset Locational data from transmitter #107908. (XLSX)

S4 Dataset Locational data from transmitter #107910. (XLSX)

S5 Dataset Locational data from transmitter #107914. (XLSX)

S6 Dataset Locational data from transmitter #107915. (XLSX)
